# Radical Cure of Hemorrhoids by Operation with Smith’s Clamps

**Published:** 1868-01

**Authors:** R. L. Walston

**Affiliations:** Paris, Illinois


ARTICLE II.

### RADICAL CURE OF HEMORRHOIDS BY OPERATION WITH SMITH'S CLAMPS.

BY R. L. WALSTON, M.D.

Mrs. O., married, age 29, mother of two children, youngest 5 years old, called at my office, January 16th, 1867, and asked professional advice and aid. She was not much emaciated, but was sallow; pulse feeble, and about 100 to the minute; was weak, dyspeptic, annoyed with tinnitus aurium, giddiness, and palpitations. She had a condition of blood akin to leucocythemia, and had suffered for three years with a most distressing hemorrhoids, aggravated by dysmenorrhea and prolapsus uteri. Each catamenia had been preceded by from two to six days' intolerable suffering, which she affirmed to be greater than that of child-birth. Her bowels were habitually constipated, for which she got the following:—Ex. coly. com. et pulvis aloes, āā grs. xx., pulvis ipecac et capsicum, āā grs. x., oil sassafras, gtts. x. M. Ft. pills 20. S. One every night. For the existing anemia and dysmccorrhea, she got, tinct. cimicifuga ℥iv., sol. cit. ferri ℥iss., sul. quinia grs. x. l. M.S., teaspoonful three times each day.

Saw her again February 1st, improvement was very marked in all particulars, except the hemorrhoids, which, unexpectedly, were not improved. I determined upon immediate operation by the use of "Smith's Clamps" for radical cure. She got full dose coly. et aloes, to be followed by castor oil if bowels were not moved. Saw her on the morning of the 2d; bowels not moved, but catamenia had come in the night about one week too soon, and without the slightest premonitory pain. During the day the bowels were well evacuated, after which the operation was performed as follows:—The patient was "chloroformed" and placed on the table in position for lithotomy. After a thorough examination of the rectum, it was ascertained that, although the aloes had greatly reduced the size of the tumors, there were four external and one internal which required removal. The tumors were found to consist of a congeries of vericose veins, surrounded by hypertrophed areolar tissue, and covered by mucous membrane, more or less altered by chronic inflammation. The internal tumor was brought down to the verge of the anus by the index finger of the left hand, and siezed by a volcella, where it was firmly held till its base could be grasped by the clamps and removed with a strong pair of scissors, and cauterized with the actual cautery, at white heat, till not a jet of hemorrhage remained, and so on till all were removed, after which the patient got large doses of morphia, with poultice to the fundament, till pain and inflammation subsided, when she again resumed the general treatment. She is now, for the first time within three years, performing all her domestic duties, and seems to enjoy perfect health.

The clamps and the cautery are the *sine qua non* for all similar cases; also in prolapsus ani they answer an admirable purpose for removing longitudinal sections from the bowel, the cure being affected by lessening its caliber in the formation of cicatrix and unyielding adhesions.

PARIS, ILLINOIS, *November 27, 1867.*

